# Deep Neuromuscular Block with Low Pressure Pneumoperitoneum in Laparoscopic Abdominal Surgeries: A Randomized Controlled Trial

**DOI:** 10.5812/aapm-150995

**Published:** 2024-10-08

**Authors:** Ahmed Mohamed Ibrahim, Mohammed Said ElSharkawy, Reda Khalil Abdelrahman, Abdallah Elabd Hassan, Mohammed Gaber Ibrahim Saad, Ismail Ahmed Elzoughari, Abdelkarem Hussini Ismail Alsayed, Asmaa Abdelbadie, Rehab Abd El Fattah Helal

**Affiliations:** 1 Department of Surgical Intensive Care and Pain Medicine, Faculty of Medicine, Tanta University, Tanta, Egypt; 2 Intensive Care and Pain Management, Faculty of Medicine, Al-Azhar University, Damietta, Egypt; 3 Intensive Care and Pain Management, Faculty of Medicine, Al-Azhar University, Cairo, Egypt; 4 Clinical Pharmacy, Department of Clinical Pharmacy, Faculty of Pharmacy, Nahda University, Beni-suef, Egypt

**Keywords:** Neuromuscular Block, Low Pressure, Postoperative Pain, Laparoscopic, Pneumoperitoneum

## Abstract

**Background:**

Postoperative pain management remains a challenge in laparoscopic abdominal surgeries.

**Objectives:**

The purpose of this research was to determine the effects of deep neuromuscular blockade (NMB) in conjunction with low-pressure pneumoperitoneum )PP) on postoperative pain, surgical parameters, and recovery outcomes.

**Methods:**

This randomized controlled double-blind study included 44 participants undergoing laparoscopic abdominal surgery. Patients were randomized equally into two groups (22 each): Group D received deep NMB, while group M received moderate NMB using cis-atracurium, through the utilization of computer-generated random numbers enclosed within sealed, opaque envelopes, following a parallel approach. Regarding deep NMB, following an initial dose of 0.15 mg/kg, a continuous infusion of 0.06 - 0.12 mg/kg/hr was administered to maintain a post-tetanic count between 1 and 2, with low PP pressure of 10 - 12 mmHg. Conversely, for moderate NMB, after the same initial dose of 0.15 mg/kg, the continuous infusion commenced upon the train-of-four count returning to 2, with the rate adjusted to sustain a count between 1 and 3, and standard PP pressure of 15 mmHg. The primary outcome was postoperative pain intensity as measured by Numerical Rating Scale (NRS) scores in the post-anesthesia care unit (PACU). The secondary outcomes included postoperative pain intensity measured by NRS scores from 2 hours to 48 hours post-surgery, time to first analgesic administration, cumulative opioid consumption within the initial 48-hour postoperative period, and patient-reported satisfaction with postoperative pain management. Statistical analysis using SPSS v26 included tests for normality (Shapiro-Wilks), with parametric data analyzed by *t*-test, non-parametric data by Mann-Whitney, and qualitative data by chi-square/Fisher's test.

**Results:**

Group D experienced a considerably longer time until the first analgesic rescue compared to group M (9.82 ± 1.5 hours vs. 7.23 ± 1.19 hours, P < 0.001). Morphine consumption in the first 24 hours was lower in Group D (10.77 ± 1.51 mg vs. 13.09 ± 1.74 mg, P < 0.001). At 6, 8, and 12 hours postoperatively, group D exhibited significantly lower pain scores (P < 0.05). Surgical duration, surgical field quality, complication rates, and patient satisfaction were comparable between groups.

**Conclusions:**

Deep NMB combined with low-pressure PP provided superior postoperative analgesia without compromising surgical field quality or increasing complications in laparoscopic abdominal surgeries.

## 1. Background

Inadequate acute pain management following surgical procedures is associated with a myriad of adverse effects, such as higher rates of morbidity, decreased ability to engage in physical activities, reduced quality of life, delayed recovery, prolonged use of opioid medications both during and after hospitalization, and escalated healthcare costs (1). Moreover, early postoperative pain appears to be a precipitating factor for the development of persistent pain syndromes that may persist for months after the operation in a substantial proportion of patients (1).

One strategy proposed to mitigate postoperative pain involves reducing the (PP) pressure during laparoscopic procedures. While the pathophysiological mechanisms underlying intraoperative PP-induced pain are not fully clarified, a compelling theory suggests that the carbon dioxide gas used to maintain intra-abdominal pressure may stretch the peritoneum and irritate the diaphragm, thereby inducing pain (2).

Concurrently, neuromuscular blocking (NMB) agents are essential components of general anesthesia, and emerging evidence suggests that significant NMB throughout anesthesia can effectively decrease the severity of pain following surgery and enhance surgical visibility (3, 4). Deep NMB during abdominal procedures has been linked to several benefits, including lower abdominal pressures, reduced postoperative pain and opioid requirements, and decreased intraoperative bleeding (5, 6). With deep NMB, lower insufflation pressures may be possible without sacrificing the surgeon's view of the operating field. However, the widespread adoption of deep NMB has been limited by the absence of reliable and rapid recovery provided by traditional NMB reversal agents like neostigmine or spontaneous recovery (7).

Laparoscopic abdominal surgeries offer notable benefits over open procedures, including less postoperative pain, shorter hospital stays, improved cosmetic results, and higher patient satisfaction. However, these procedures still present challenges. Injecting carbon dioxide into the peritoneal cavity to induce PP increases the pressure inside the abdomen, which can lead to cardiovascular, pulmonary, and splanchnic perfusion alterations (8, 9).

## 2. Objectives

The purpose of this research was to determine the effects of deep NMB in conjunction with low-pressure pneumoperitoneum )PP) on postoperative pain, surgical parameters, and recovery outcomes.

## 3. Methods

A controlled randomized trial was conducted on 44 participants (aged 18 to 65 years), both sexes, with an American Society of Anesthesiologists physical status of I-III, who were scheduled for laparoscopic abdominal surgery at Tanta University Hospitals, Egypt, from October 2023 to March 2024. The study received approval from the institutional ethical committee and was registered on ClinicalTrials.gov (ID: NCT06242262). Informed written consent was obtained from the patients' relatives.

The exclusion criteria included allergies to cis-atracurium or neostigmine, contraindications for neostigmine use, a history of neuromuscular, kidney, or liver disease, previous abdominal surgeries, preoperative hyperalgesia, peripheral neuropathy due to diabetes, chronic analgesic treatment or substance abuse, and a Body Mass Index (BMI) of 35 kg/m² or more.

Preoperatively, all participants fasted for 8 hours and underwent the collection of medical histories, clinical assessments, and standard laboratory tests. The trial design and pain score scale were explained during the preoperative anesthesia visit.

Intraoperatively, standard general anesthesia techniques were employed, with monitoring conducted through pulse oximetry, temperature assessment, non-invasive blood pressure measurement, electrocardiogram, and capnography.

### 3.1. Randomization and Blindness

Participants were randomly allocated into two equivalent groups using computer-generated random numbers enclosed within sealed, opaque envelopes, following a parallel approach. Group D (n = 22) received deep NMB using cis-atracurium, while group M (n = 22) received moderate NMB with the same drug. Both patients and outcome evaluators were kept unaware of the group assignments. Prior to the administration of general anesthesia, a separate anesthesiologist, who was not involved in data collection or analysis, performed the blocking procedure.

Outcome assessors remained blinded throughout the study period by ensuring they had no access to the anesthesia records or operating room. All neuromuscular monitoring equipment was removed before the assessors entered the post-anesthesia care unit )PACU). Patient charts were specifically prepared to exclude any information that could reveal group assignments.

To prevent inadvertent unblinding due to different neuromuscular blockade )NMB) levels, the surgical team was instructed not to discuss muscle relaxation or surgical field conditions in the presence of outcome assessors. The anesthesiologist managing the NMB used a screen to conceal the neuromuscular monitoring display from other operating room personnel.

While deep and moderate NMB can potentially result in visible differences in muscle relaxation, our use of standardized surgical techniques and careful management of PP pressure helped minimize any observable differences between groups. The surgical team reported no consistent visible differences in muscle relaxation or surgical field conditions that could have compromised blinding.

To assess the integrity of blinding, we asked both patients and outcome assessors to guess their group assignment at the end of the study. The results indicated that guesses were no better than chance (52% correct for patients, 54% for assessors), suggesting that blinding was successfully maintained throughout the study period.

The induction of general anesthesia was accomplished by administering intravenous (IV) propofol at a dose of 2 - 2.5 mg/kg, along with IV fentanyl at a dose of 1 μg/kg. This was followed by the administration of IV cis-atracurium at a dose of 0.15 mg/kg for endotracheal intubation and a PP pressure of 15 mmHg. The patient was maintained under anesthesia using isoflurane (1 - 1.5%) and 50% oxygen. Additionally, a continuous infusion of cis-atracurium at a rate of 0.06 - 0.12 mg/kg/h was administered to maintain the desired level of muscle relaxation with a PP pressure of 10 - 12 mmHg. Entropy monitoring was utilized to adjust the doses of fentanyl and isoflurane. A tidal volume of 6 - 8 mL/kg and an end-tidal CO₂ pressure of 35 - 45 mmHg were maintained for volume control mode ventilation. Continuous monitoring was performed to ensure that core temperatures remained above 36°C.

After 15 minutes of tracheal intubation, the rate of the cis-atracurium pump was adjusted in Group D so that the post-tetanic count (PTC) remained between one and two. In group M, the cis-atracurium pump was started when the train-of-four (TOF) count returned to 2, with the rate adjusted to maintain the TOF count between 1 and 3. The cis-atracurium infusion was temporarily stopped in both groups if muscle relaxation deepened beyond the predefined levels until it returned to the target range. About thirty minutes before the procedure was completed, the cis-atracurium infusion was discontinued.

Upon completion of surgery, every patient had their muscle relaxant monitoring mode changed to TOF mode. Whenever the TOF count rose above 70% or returned to 2, 1 mg of neostigmine and 0.5 mg of atropine were administered. The process of removing the endotracheal tube was carried out once the TOF count reached 90% and the patient demonstrated the ability to comply with commands, such as opening their eyes and shaking their hand, as assessed by the anesthesiologist. High-flow oxygen was administered via a mask after extubation.

Postoperatively, the individuals were admitted to the PACU for routine monitoring once their blood oxygen saturation level remained consistently above 95%. A standardized analgesic regimen of paracetamol (1 g every 6 hours) was prescribed, with IV morphine (3 mg) as rescue analgesia if the Numerical Rating Scale (NRS) for pain exceeded 3.

When assessing postoperative abdominal pain, researchers used the NRS (0 = no pain, 10 = worst pain) assessed by nursing staff unrelated to the study, at the PACU and at 4, 6, 12, 24, and 48 hours postoperatively. The time at which the first rescue analgesic was administered, as well as the total amount of pain relief medication consumed within the first 24 and 48 hours, were documented. Additionally, the quality of the surgical field, length of surgery, heart rate (HR), mean arterial pressure (MAP), postoperative complications such as bradycardia, hypotension, nausea, vomiting, and patient satisfaction on a 5-point scale were recorded.

### 3.2. Size of Sample Calculation

The calculation of sample size was performed using G*Power 3.1.9.2 software (Universitat Kiel, Germany). Based on earlier research (8), the postoperative pain score on the NRS in the PACU, which was the primary outcome measure, reported a mean ± standard deviation of 2.3 ± 0.6 for deep NMB and 2.9 ± 0.3 for moderate NMB. The assessment of the sample size was based on the following parameters: An effect size of 1.264, a 95% confidence level, a statistical power of 95%, a 1:1 group ratio, and the addition of four cases per group to account for potential dropouts. Consequently, the recruitment goal was established at 22 patients per group.

### 3.3. Statistical Analysis

The statistical analysis was conducted using SPSS V26 (IBM Inc., Chicago, IL, USA). The normality of the data distribution was assessed using the Shapiro-Wilk test and histograms. Quantitative parametric variables were expressed as mean and standard deviation and compared between the groups using the unpaired Student's *t*-test. Quantitative non-parametric data were presented as median and interquartile range (IQR) and analyzed using the Mann-Whitney test. Qualitative variables were presented as frequency and percentage (%) and analyzed using the chi-square test or Fisher's exact test as appropriate. A two-tailed P-value of < 0.05 was considered statistically significant.

## 4. Results

**Figure 1.**
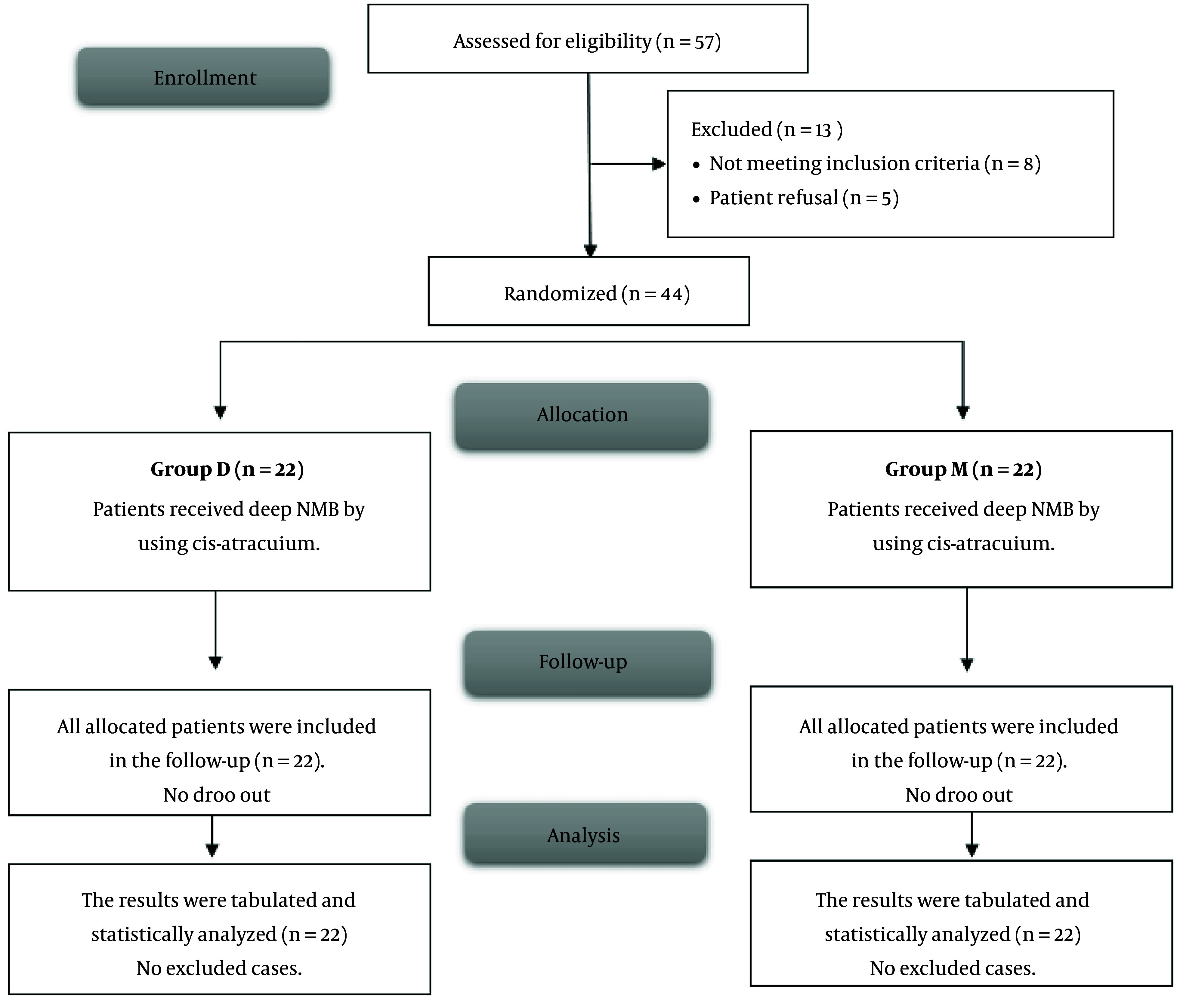
CONSORT flowchart of the enrolled patients

In this investigation, 57 patients were initially screened for eligibility. Of these, 8 did not meet the criteria, and 5 declined to participate. The remaining eligible patients were then randomly assigned to two groups, each comprising 22 patients. Subsequently, all patients assigned to their respective groups were closely followed up and subjected to statistical analysis (Figure 1). 

The demographic data revealed no significant differences between the two study groups (Table 1). The duration of surgery and the quality of the surgical field were similar across groups, with increased intra-abdominal pressure. The time to the first request for rescue analgesia was significantly prolonged in group D compared to group M (9.82 ± 1.5 hours vs. 7.23 ± 1.19 hours, P < 0.001), suggesting improved postoperative pain management. Correspondingly, the total dose of morphine consumption in the first 24 hours was lower in group D compared to group M (10.77 ± 1.51 mg vs. 13.09 ± 1.74 mg, P < 0.001), further corroborating the superior analgesic efficacy observed in the intervention group. The NRS scores for postoperative pain did not differ significantly between the groups in the PACU or at 1, 2, 4, 18, 24, 36, and 48 hours after surgery. However, at 6, 8, and 12 hours postoperatively, group D exhibited significantly lower NRS scores compared to group M (P < 0.05), indicating better pain control during this critical period (Table 2). 

**Table 1.** Demographic Data of the Studied Groups ^a^

Variables	Group D; (n = 22)	Group M; (n = 22)	P-Value
**Age (y)**	51.5 ± 14.43	54.18 ± 13.16	0.523
**Gender**			0.540
Male	14 (63.64)	12 (54.55)	
Female	8 (36.36)	10 (45.45)	
**Weight (kg)**	68.64 ± 7.79	71.45 ± 9.99	0.303
**Height **	167.41 ± 6.9	166.36 ± 5.8	0.589
**BMI (kg/m** ^**2**^ **)**	24.6 ± 3.28	25.77 ± 2.94	0.217
**ASA physical status**			0.553
I	12 (54.55)	14 (63.64)	
II	8 (36.36)	7 (31.82)	
III	2 (9.09)	1 (4.55)	

Abbreviations: BMI, Body Mass Index; ASA, American Society of Anesthesiologists.

^a^ Values are expressed as mean ± SD or No. (%).

**Table 2.** Duration of Surgery and Quality of Surgical Field for Increase Intra-abdominal Pressure, Time to First Request of Rescue Analgesia, Total Dose of Morphine Consumption in the First 24 Hours and Numerical Rating Scale of the Studied Groups

Variables	Group D; (n = 22)	Group M; (n = 22)	P-Value
**Duration of surgery (min)**	81.82 ± 14.76	84.77 ± 19.67	0.576
**Quality of surgical field for increase intra-abdominal pressure**	2.32 ± 0.72	2.73 ± 0.77	0.075
**Time to first request of rescue analgesia (h)**	9.82 ± 1.5	7.23 ± 1.19	< 0.001 ^b^
**Total dose of morphine consumption in the first 24 hours (mg)**	10.77 ± 1.51	13.09 ± 1.74	< 0.001 ^b^
**NRS**			
PACU	0 (0 - 1)	0 (0 - 1)	0.359
1 h	0 (0 - 1)	0 (0 - 1)	0.136
2 h	2 (1 - 2)	2 (1 - 2)	0.760
4 h	2 (1 - 2)	2 (1.25 - 2)	0.116
6 h	2 (1 - 2)	2 (1.25 - 2)	0.042 ^b^
8 h	2 (1 - 3.5)	3 (2.25 - 4.5)	0.010 ^b^
12 h	2 (1 - 2.75)	3.5 (2 - 4.75)	0.020 ^b^
18 h	4 (3 - 4)	4 (3 - 5)	0.207
24 h	2 (1 - 2.75)	3.5 (2 - 4.75)	0.513
36 h	4 (3 - 4)	4 (3 - 5)	0.311
48 h	4 (3.25 - 4)	4 (4 - 4.75)	0.570

Abbreviations: NRS, Numerical Rating Scale; PACU, post-anesthesia care unit.

^a^ Values are expressed as mean ± SD or median (IQR).

^b^ Significant when P value ≤ 0.05.

**Figure 2.**
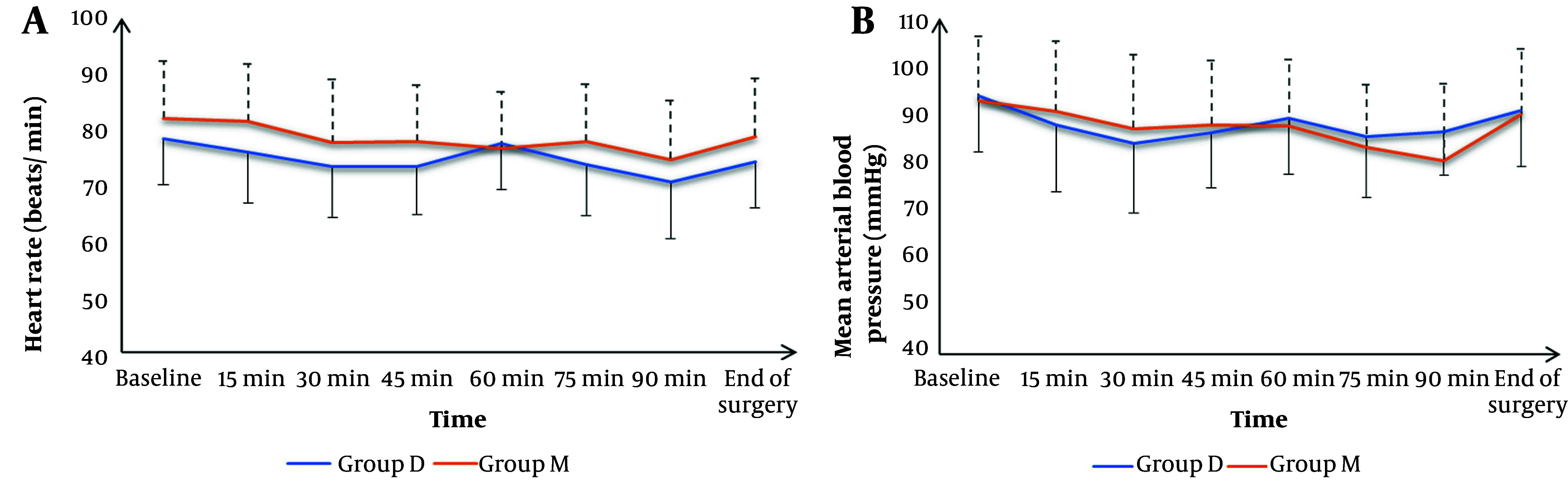
A, heart rate; and B, mean blood pressure of the studied groups

Heart rate and MAP were insignificantly different at baseline, and at 15, 30, 45, 60, 75, 90 minutes, and at the end of surgery between both groups (Figure 2). The frequency of complications, including hypotension, bradycardia, and postoperative nausea and vomiting (PONV), was statistically insignificant among the two groups. Similarly, patient satisfaction levels did not differ significantly (Table 3). 

**Table 3.** Complications and Patients’ Satisfaction of Studied Groups ^a^

Variables	Group D (n = 22)	Group M (n = 22)	P-Value
**Complications**			
Bradycardia	3 (13.64)	2 (9.09)	1
Hypotension	5 (22.73)	3 (13.64)	0.698
PONV	2 (9.09)	4 (18.18)	0.664
**Patients’ satisfaction**			0.567
Extremely satisfied	7 (31.82)	4 (18.18)	
Satisfied	10 (45.45)	9 (40.91)	
Neither satisfied nor unsatisfied	4 (18.18)	7 (31.82)	
Unsatisfied	1 (4.55)	2 (9.09)	
Extremely dissatisfied	0 (0)	0 (0)	

Abbreviation: PONV, postoperative nausea and vomiting.

^a^ Values are expressed as No. (%).

## 5. Discussion

The role of deep NMB and low-pressure PP in mitigating postoperative pain following laparoscopic abdominal surgery has been an area of active investigation (2, 6-8, 10, 11). The present study did not find a significant difference in surgery length between the deep and moderate NMB groups. Similarly, Kim et al. (8) and Koo et al. (11) did not find significant differences in surgery duration between the two groups.

Regarding the quality of the surgical field during increased intra-abdominal pressure, the present study discovered that neither group differed significantly from the other. This finding contrasts with several previous studies, including Kim et al. (8), Bruintjes et al. (12), and Reijnders-Boerboom et al. (13), which noted a marked improvement in surgical outcomes with deep NMB. However, it is important to consider that the quality of the surgical field can be influenced by various factors, such as the surgeon's experience, the type of surgery, and the specific surgical techniques employed.

Hemodynamics (HR and MAP) were insignificantly different between the two groups throughout the surgical procedure. This finding aligns with previous studies by Kim et al. (8) and Honing et al. (14), which reported no significant differences in these vital signs between deep and moderate NMB groups. However, Oh et al. (15) observed lower HR and MAP in the deep NMB group during lumbar spinal surgery, suggesting potential benefits in specific surgical contexts.

A notable finding of this research was that the deep NMB group experienced a delay in the first request for rescue analgesia compared to the moderate NMB group (9.82 ± 1.5 hours vs. 7.23 ± 1.19 hours, P < 0.001). Correspondingly, the quantity of morphine taken in the first 24 hours was significantly lower in the deep NMB group (10.77 ± 1.51 mg vs. 13.09 ± 1.74 mg, P < 0.001). These results corroborate the findings of Kim et al. (8) and Tang et al. (16), who reported reduced postoperative opioid requirements and improved analgesia in individuals receiving deep NMB. The enhanced pain management observed in the deep NMB group could be attributed to the improved surgical conditions, potentially leading to less tissue trauma and inflammation.

Interestingly, while there was no significant difference in the NRS pain scores between groups in the PACU or at most time points, the deep NMB group had significantly lower scores at 6, 8, and 12 hours postoperatively. This finding aligns with the meta-analysis by Raval et al. (7), which reported reduced severity of pain in the PACU with deep NMB following surgery. The improved pain control during this critical early postoperative period could contribute to enhanced patient satisfaction and faster recovery. In contrast, Honing et al. (14) reported NRS scores of approximately 2.9 for moderate NMB and ~3.2 for deep NMB in laparoscopic renal surgery with sevoflurane anesthesia.

The incidence of complications, including bradycardia, hypotension, and PONV, was statistically insignificant among groups. This observation is consistent with prior research, such as that by Koo et al. (9) and Arumugaswamy et al. (17), which found no differences in adverse events between deep and moderate NMB groups. However, Oh et al. (15) and Hu et al. (18) reported lower occurrences of PONV and hypotension in the deep NMB group, suggesting potential benefits in specific surgical contexts.

Patient satisfaction levels were also comparable between the two cohorts in the current investigation. While this finding contrasts with the study by Koo et al. (19), which reported higher patient satisfaction scores with deep NMB, it is essential to note that patient satisfaction can be influenced by various factors beyond surgical outcomes, such as preoperative expectations, communication, and overall hospital experience.

The findings from other studies further support the possible advantages of deep NMB during laparoscopic procedures. Barrio et al. (20) indicated that deep NMB significantly increased the intra-abdominal volume of CO_2_ insufflated compared to moderate NMB, facilitating a better surgical field during PP establishment. Koo et al. (9) reported shorter operation times and a decreased rate of intra-abdominal pressure increase to preserve optimal surgical conditions with deep NMB during laparoscopic cholecystectomy. Additionally, Koo et al. (19) and Koo et al. (11) found that deep NMB was associated with better surgical conditions and less intraoperative movement during laparoscopic colorectal and gastric surgeries, respectively.

One limitation of this research is the sample size; while adequate for the primary outcome, it may have been underpowered to detect differences in secondary outcomes or rare adverse events. Additionally, its single-center design may restrict the applicability of the results to different types of surgical procedures and patient groups.

### 5.1. Conclusions

The deep NMB combined with low-pressure PP provided superior postoperative analgesia, as evidenced by the prolonged time to the first rescue analgesic request and reduced morphine use in the first 24 hours compared to moderate NMB. Importantly, these analgesic benefits were achieved without compromising surgical field quality or increasing complications. Furthermore, the current study supports the use of deep NMB as a valuable adjunct for enhancing postoperative pain management in laparoscopic surgical procedures related to the abdomen.

## Data Availability

The data presented in this study are uploaded during submission as a supplementary file and are openly available for readers upon request.

## Clinical Trial Registration Code:

NCT06242262.

## References

[1] Gan TJ (2017). Poorly controlled postoperative pain: prevalence, consequences, and prevention.. J Pain Res..

[2] Chang W, Yoo T, Cho WT, Cho G (2021). Comparing postoperative pain in various pressure pneumoperitoneum of laparoscopic cholecystectomy: a double-blind randomized controlled study.. Ann Surg Treat Res..

[3] Liu S, He B, Deng L, Li Q, Wang X (2023). Does deep neuromuscular blockade provide improved perioperative outcomes in adult patients? A systematic review and meta-analysis of randomized controlled trials.. PLoS One..

[4] Prabhakar NK, Chadwick AL, Nwaneshiudu C, Aggarwal A, Salmasi V, Lii TR (2022). Management of Postoperative Pain in Patients Following Spine Surgery: A Narrative Review.. Int J Gen Med..

[5] Edinoff AN, Fitz-Gerald JS, Holland KAA, Reed JG, Murnane SE, Minter SG (2021). Adjuvant Drugs for Peripheral Nerve Blocks: The Role of NMDA Antagonists, Neostigmine, Epinephrine, and Sodium Bicarbonate.. Anesth Pain Med..

[6] Li HX, Xu X, Tan PX, Wang TH, Li BL, Zheng H (2024). [The effect of deep neuromuscular block combined with low pneumoperitoneum pressure on postoperative pain in patients undergoing laparoscopic radical colorectal surgery].. Zhonghua Yi Xue Za Zhi..

[7] Raval AD, Deshpande S, Rabar S, Koufopoulou M, Neupane B, Iheanacho I (2020). Does deep neuromuscular blockade during laparoscopy procedures change patient, surgical, and healthcare resource outcomes? A systematic review and meta-analysis of randomized controlled trials.. PLoS One..

[8] Kim MH, Lee KY, Lee KY, Min BS, Yoo YC (2016). Maintaining Optimal Surgical Conditions With Low Insufflation Pressures is Possible With Deep Neuromuscular Blockade During Laparoscopic Colorectal Surgery: A Prospective, Randomized, Double-Blind, Parallel-Group Clinical Trial.. Medicine (Baltimore)..

[9] Koo BW, Oh AY, Seo KS, Han JW, Han HS, Yoon YS (2016). Randomized Clinical Trial of Moderate Versus Deep Neuromuscular Block for Low-Pressure Pneumoperitoneum During Laparoscopic Cholecystectomy.. World J Surg..

[10] Barrio J, Errando CL, Garcia-Ramon J, Selles R, San Miguel G, Gallego J (2017). Influence of depth of neuromuscular blockade on surgical conditions during low-pressure pneumoperitoneum laparoscopic cholecystectomy: A randomized blinded study.. J Clin Anesth..

[11] Koo BW, Oh AY, Ryu JH, Lee YJ, Han JW, Nam SW (2019). Effects of deep neuromuscular blockade on the stress response during laparoscopic gastrectomy Randomized controlled trials.. Sci Rep..

[12] Bruintjes MH, van Helden EV, Braat AE, Dahan A, Scheffer GJ, van Laarhoven CJ (2017). Deep neuromuscular block to optimize surgical space conditions during laparoscopic surgery: a systematic review and meta-analysis.. Br J Anaesth..

[13] Reijnders-Boerboom G, van Helden EV, Minnee RC, Albers KI, Bruintjes MHD, Dahan A (2021). Deep neuromuscular block reduces the incidence of intra-operative complications during laparoscopic donor nephrectomy: a pooled analysis of randomized controlled trials.. Perioper Med (Lond)..

[14] Honing GHM, Martini CH, Olofsen E, Bevers RFM, Huurman VAL, Alwayn IPJ (2021). Deep neuromuscular block does not improve surgical conditions in patients receiving sevoflurane anaesthesia for laparoscopic renal surgery.. Br J Anaesth..

[15] Oh SK, Kwon WK, Park S, Ji SG, Kim JH, Park YK (2019). Comparison of Operating Conditions, Postoperative Pain and Recovery, and Overall Satisfaction of Surgeons with Deep vs. No Neuromuscular Blockade for Spinal Surgery under General Anesthesia: A Prospective Randomized Controlled Trial.. J Clin Med..

[16] Tang X, Wu Y, Chen Q, Xu Y, Wang X, Liu S (2023). Deep Neuromuscular Block Attenuates Chronic Postsurgical Pain and Enhances Long-Term Postoperative Recovery After Spinal Surgery: A Randomized Controlled Trial.. Pain Ther..

[17] Arumugaswamy PR, Chumber S, Rathore YS, Maitra S, Bhattacharjee HK, Bansal VK (2024). Low-pressure pneumoperitoneum with deep neuromuscular blockade versus standard pressure pneumoperitoneum in patients undergoing laparoscopic cholecystectomy for gallstone disease: a non-inferiority randomized control trial.. Surg Endosc..

[18] Hu G, Shao W, Chen Z, Li B, Xu B (2023). Deep neuromuscular block attenuates intra-abdominal pressure and inflammation and improves post-operative cognition in prostate cancer patients following robotic-assisted radical prostatectomy.. Int J Med Robot..

[19] Koo BW, Oh AY, Na HS, Lee HJ, Kang SB, Kim DW (2018). Effects of depth of neuromuscular block on surgical conditions during laparoscopic colorectal surgery: a randomised controlled trial.. Anaesthesia..

[20] Barrio J, Errando CL, San Miguel G, Salas BI, Raga J, Carrion JL (2016). Effect of depth of neuromuscular blockade on the abdominal space during pneumoperitoneum establishment in laparoscopic surgery.. J Clin Anesth..

